# Forecasting effects of angler harvest and climate change on smallmouth bass abundance at the southern edge of their range

**DOI:** 10.1371/journal.pone.0202737

**Published:** 2018-08-20

**Authors:** Christopher R. Middaugh, Daniel D. Magoulick

**Affiliations:** 1 Arkansas Cooperative Fish & Wildlife Research Unit, Department of Biological Sciences, University of Arkansas, Fayetteville, Arkansas, United States of America; 2 U.S. Geological Survey, Arkansas Cooperative Fish & Wildlife Research Unit, Department of Biological Sciences, University of Arkansas, Fayetteville, Arkansas, United States of America; Texas A&M University, UNITED STATES

## Abstract

Climate change will affect stream systems in numerous ways over the coming century. Globally, streams are expected to experience changes in temperature and flow regime. Previous work has indicated that these changes will likely affect fish distributions, but little work has been conducted examining population level effects of climate change on warmwater fish at the warmest portion of their range. We model several potential climate change-related stressors and the resulting effects on smallmouth bass *Micropterus dolomieu* populations in the Buffalo National River, Arkansas, USA, located near the southern extent of smallmouth bass range. Smallmouth bass are a popular recreational fish in the region and angler harvest likely contributes substantially to annual mortality. We created a simulation model parameterized with data collected from the Buffalo National River to evaluate the relative importance of climate stressors and angler harvest on smallmouth bass populations. Our simulations suggest that increases in springtime temperature and reductions in river discharge during the spawning period could increase recruitment, resulting in increases in adult abundance (8% higher). However, when increased flooding and drought probabilities are considered, our model indicates the Buffalo National River could experience large reductions in adult smallmouth bass abundance (≥50% decline) and increased probability of extinction compared to present levels. Simulations showed that harvest reduction could be a viable strategy to reduce the negative effects of climate change, but that even with complete closure of harvest, smallmouth bass population levels would still be well below present abundance (46% lower than present). Efforts to reduce flooding and drought effects related to climate change in the Buffalo National River could help offset the predicted reduction in the smallmouth bass population.

## Introduction

The anticipated effects of climate change on aquatic ecosystems globally will be complex [1]-[2] and are likely to result in increased temperature, decreased dissolved oxygen, increased toxicity of pollutants, and changes in hydrologic regimes [3]. Stream fishes may be particularly susceptible to the effects of climate change. For example, climate change could affect stream fishes through changes in stream discharge patterns such as increased flooding chances [4]. Spring floods can lead to failed year classes of fishes in streams (e.g., smallmouth bass *Micropterus dolomieu*, [5]; Salmonids, [6]; shoal bass *Micropterus cataractae*, [7]). Flooding can affect smallmouth bass year class strength through nest destruction and fry displacement [8]-[10]. Flooding can also lead to mortality through rapid changes in water temperature [11]. Timing of flooding can be important as the size of the fry can determine the response to the flood event [9]-[10]. Though chances of flooding could increase due to climate change, in some regions, climate change is also expected to result in an overall reduction in precipitation which could lead to lower mean discharge levels [12]. Recruitment of fish in many stream systems can be positively affected by low discharge levels during the spawning and rearing period which often enhances year class strength [5], [11], [13]-[14]. Water temperature will also be affected by climate change and can influence growth and survival of stream fishes [15–17]. Changes in stream temperature could also lead to range restrictions or expansions [18]. Because of the many potential effects of climate change on stream systems, it can be difficult to anticipate how stream fishes will be affected.

Harvest is also an important factor structuring stream fish populations. For example, harvest of stream black bass is an important contributor to mortality in streams open to exploitation (e.g., Suwannee bass and largemouth bass, [19]; smallmouth bass, [20]-[21]). Climate change will likely make managing harvested fisheries more complex and harvest regulations will need to be adapted as fish populations change [22]-[23]. High harvest levels, when combined with climate and anthropogenic related stressors, could lead to population declines [24]. As the world population grows, it is likely that fishing pressure will increase, potentially resulting in increased harvest levels that will need to be managed in conjunction with changing climate stressors.

Smallmouth bass are a warm-water riverine species broadly distributed throughout North America. The Ozark-Ouachita Interior Highlands of Arkansas lie at the southern extent of smallmouth bass native range. The southern region of the United States is projected to warm 3–4°C by the year 2050 [12]. Along with the temperature change is an expected change in precipitation, including more extreme events leading to higher stochasticity and potentially an increase in flooding frequency and severity [25] even though average annual precipitation is projected to decrease slightly in parts of the southern region [12]. Precipitation patterns are likely to change seasonally and a decrease in precipitation is anticipated in the summer months [1], potentially leading to more severe and longer drought conditions [26]. At this southern range extent, smallmouth bass populations may be vulnerable to population declines due to climate change as summer temperatures will likely reach levels that will affect growth and survivorship [27]-[28], especially during summer drought conditions which are common in Arkansas streams and rivers [29]-[30]. Smallmouth bass are at risk of being outcompeted in some lotic habitats by largemouth bass and spotted bass, which both have a competitive advantage over smallmouth bass at higher temperatures [31].

To investigate the relative effects of angler harvest and climate change on the smallmouth bass population in the Buffalo National River, Arkansas (hereafter, Buffalo River) we created an age-structured population model to simulate various climate and harvest scenarios. The structure for the model was based on previous work comparing land use and climate change effects on lotic smallmouth bass in an Illinois river [13]. We used empirical data collected in the Buffalo River to create a Ricker recruit-spawner model modified with environmental parameters which was subsequently used to predict annual recruitment of age-0 smallmouth bass within a stage structured simulation model. Our objective was to compare the effects of climate change stressors (i.e., flooding and drought) and harvest mortality on smallmouth bass. We hypothesized that changing climate conditions would negatively affect smallmouth bass populations. In addition, we hypothesized that if current levels of harvest were maintained or increased in future climate scenarios, then smallmouth bass populations would further decline and extinction probability would increase.

## Materials and methods

### Empirical smallmouth bass data

**Fig 1:**
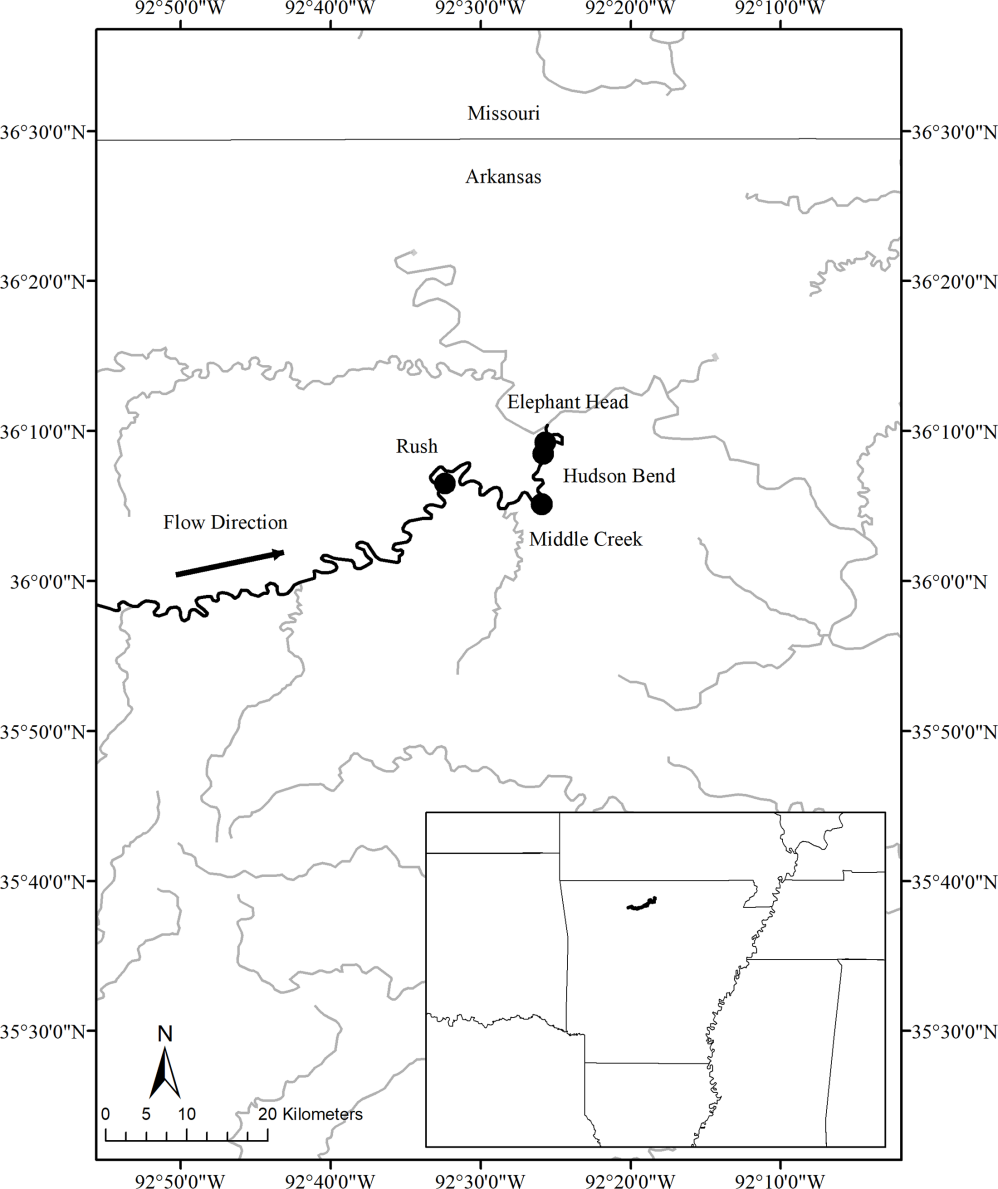
Sites in the Buffalo River, Arkansas. Map of the Buffalo River, Arkansas and the sampling locations.

The Buffalo River originates in the Boston Mountains region of Arkansas, USA and flows 238 km before entering the White River with a drainage area of 3,465 km^2^. Beginning in 1972, the National Park Service has protected approximately 90% of the river length. The Arkansas Game and Fish Commission sampled smallmouth bass in the Buffalo River twelve years between 1992 and 2012 using boat electroshocking (Fig 1). Sites were sampled a single time each year and during each sample fish were measured to the nearest millimeter and weighed to the nearest gram. We selected a subset of data from sites sampled at least three years in the month of October, and during nighttime boat electrofishing. This left 15 samples, collected from four different sites over the course of six years (Table 1). The sites were all located in the lower Buffalo River with approximately 24 miles between the most upstream (Rush) and the farthest downstream site (Elephant Head; Fig 1). Specific habitat data for each sample was not collected, however, the lower reach of the Buffalo River is characterized by large, deep pools with gravel and boulder substrate, separated by relatively short riffles and runs. We selected only sites sampled during October in order to reduce bias associated with small age-0 fish recruitment to the sampling gear and also because autumn can be a reliable indicator of year-class strength for lotic smallmouth bass [5]. We then removed any outliers by examining fish length and weight data. We did this by calculating relative weights (Wr) for each fish > 150 mm [32] and removing any fish with an extreme Wr (Wr < 55 or > 145).

**Table 1:** Empirical smallmouth bass data. Data collected by the Arkansas Game and Fish Commission in the Buffalo River, AR. Number of age-0 and adult smallmouth bass columns show ranges of counts of fish designated as those age groups.

Site	Years sampled	Num. of Age-0 (<151–176 mm)	Num. of Adults (≥250 mm)
Rush	2008–2012	5–49	43–182
Middle Creek	2008–2010	4–32	40–116
Elephant Head	2006, 2008, 2012	6–40	13–73
Hudson Bend	2006, 2011, 2012	6–34	50–52

We next created length histograms for each site to estimate the length cutoff between age-0 and older fish in order to determine the number of age-0 fish collected during each sampling event. This length cutoff varied by site and by year (Table 1). We assume that fish age-3+ are mature based on previous work in the Buffalo River [33] and we determined the number of adults at each sampling event by counting the number of fish ≥ 225 mm based on estimates for age-3 smallmouth bass in the Buffalo River [34]. Counts of age-0 and adult fish were then standardized by dividing by sampling effort (minutes of shocking time) for each sample. These standardized catch-per-unit-effort (CPUE) data were then used in subsequent analyses.

The fish used in this study were collected by AGFC personnel using standardized methods for monitoring purposes prior to the authors' involvement in the study. No fish were reported as being harmed or euthanized as part of data collection. IACUC approval was not applied for, but AGFC biologists follow appropriate animal handling and use procedures.

### Environmental data

**Fig 2:**
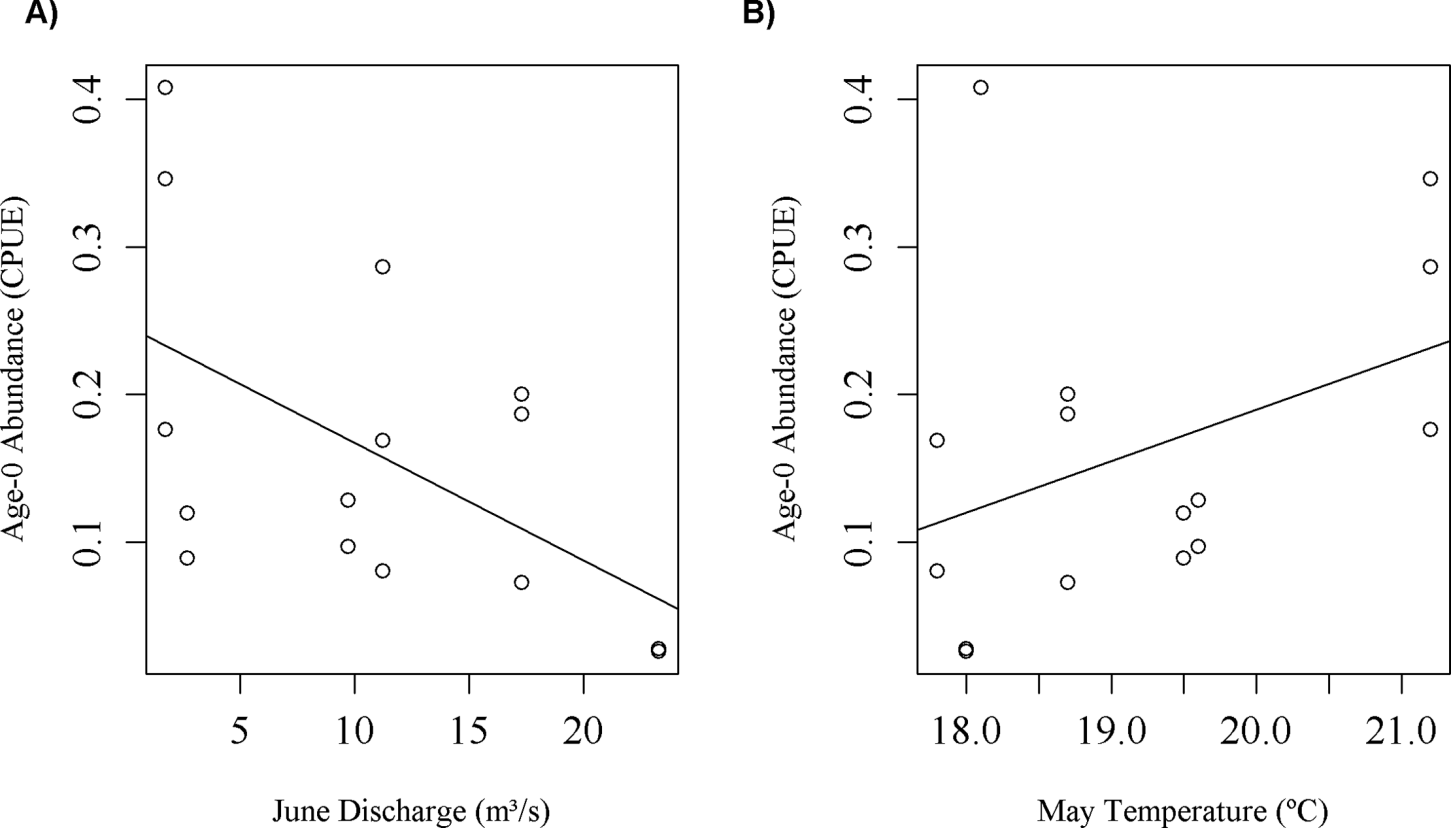
Relationships between age-0 smallmouth bass abundance and environmental factors. Relationship between June discharge (A) and May temperature (B) and number of age-0 smallmouth bass collected by the Arkansas Game and Fish Commission during October boat electrofishing samples. Best fit lines are shown in plots to demonstrate trends.

Environmental data for monthly mean air temperature and monthly mean river discharge were obtained in order to relate to yearly CPUE of age-0 smallmouth bass. We selected these discharge and temperature variables to relate to age-0 fish abundances as similar environmental variables are related to age-0 smallmouth bass recruitment in other river systems [5], [13], [35]-[36] and we chose to include both temperature and discharge variables in the final model as both factors are important in structuring recruitment and can be interrelated [37]. We downloaded daily discharge data from USGS gage 07056000 on the Buffalo River near St. Joe, AR. We used these data to determine mean monthly discharge and standard deviation for the years 1940–2013. Air temperature data was downloaded from long-term climate data collected by a National Oceanic and Atmospheric Agency National Center for Climate Data weather station in nearby Harrison, AR (Station USW00013971). We related mean monthly air temperature and mean monthly discharge to CPUE of age-0 smallmouth bass for each month during the spawning/rearing period (March-July) using a simple linear regression for each variable. The month with the strongest relationship based on least squares regression R^2^ value for each environmental parameter (Fig 2) was selected for use in a Ricker recruit-spawner model with environmental terms incorporated (see below). We selected the best models using R^2^ because an environmental variable was used as a response variable one at a time in linear regression models.

### Model overview

**Fig 3:**
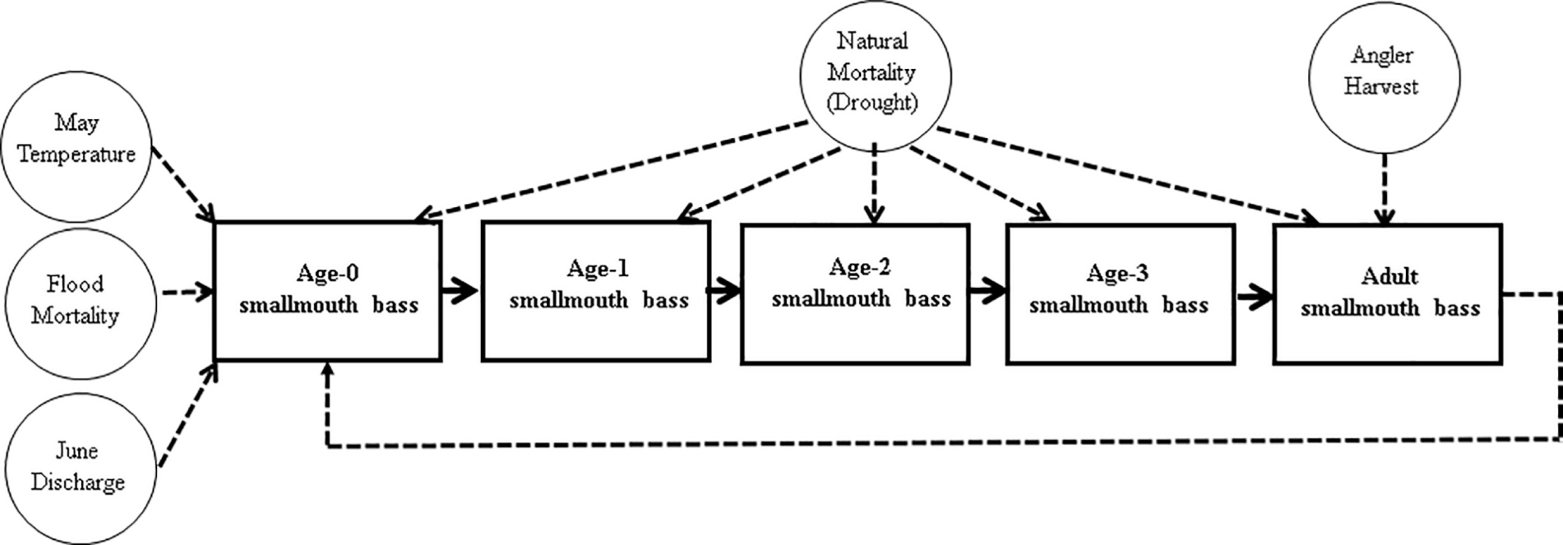
Model diagram. Conceptual diagram of the age-structured simulation model. Rectangles represent age-classes of fish and circles represent parameters affecting different age groups.

We created an age-structured smallmouth bass model with age-specific environmental effects, and harvest mortality (Fig 3). Fish age groups are set as age-0, age-1, age-2, age-3 and age-4+ to age-8 (we assume 100% mortality of age-8 fish as no fish older than this were collected by [34]. All smallmouth bass abundances are reported in units of CPUE because these are the units our empirical data is based on. Final model output examines relative changes in predicted abundances of smallmouth bass in the Buffalo River rather than absolute changes in fish abundances. The model was programmed and run in R [38].

### Age-0 fish abundance

Age-0 fish abundances in the model are determined using a Ricker recruit-spawner model with environmental terms incorporated (e.g., [13], [39]). This type of model incorporates adult densities and assumes competition among juveniles in order to predict number of recruits. We incorporate May temperature and June discharge into the model as these were the best predictors of age-0 fish abundance from empirical data. The Ricker recruit-spawner model was structured as:

CPUE(age0)=CPUE(adults)*exp(a−(b*CPUE(adults))+(c*MayTemp)+(d*JuneDischarge))

**Fig 4:**
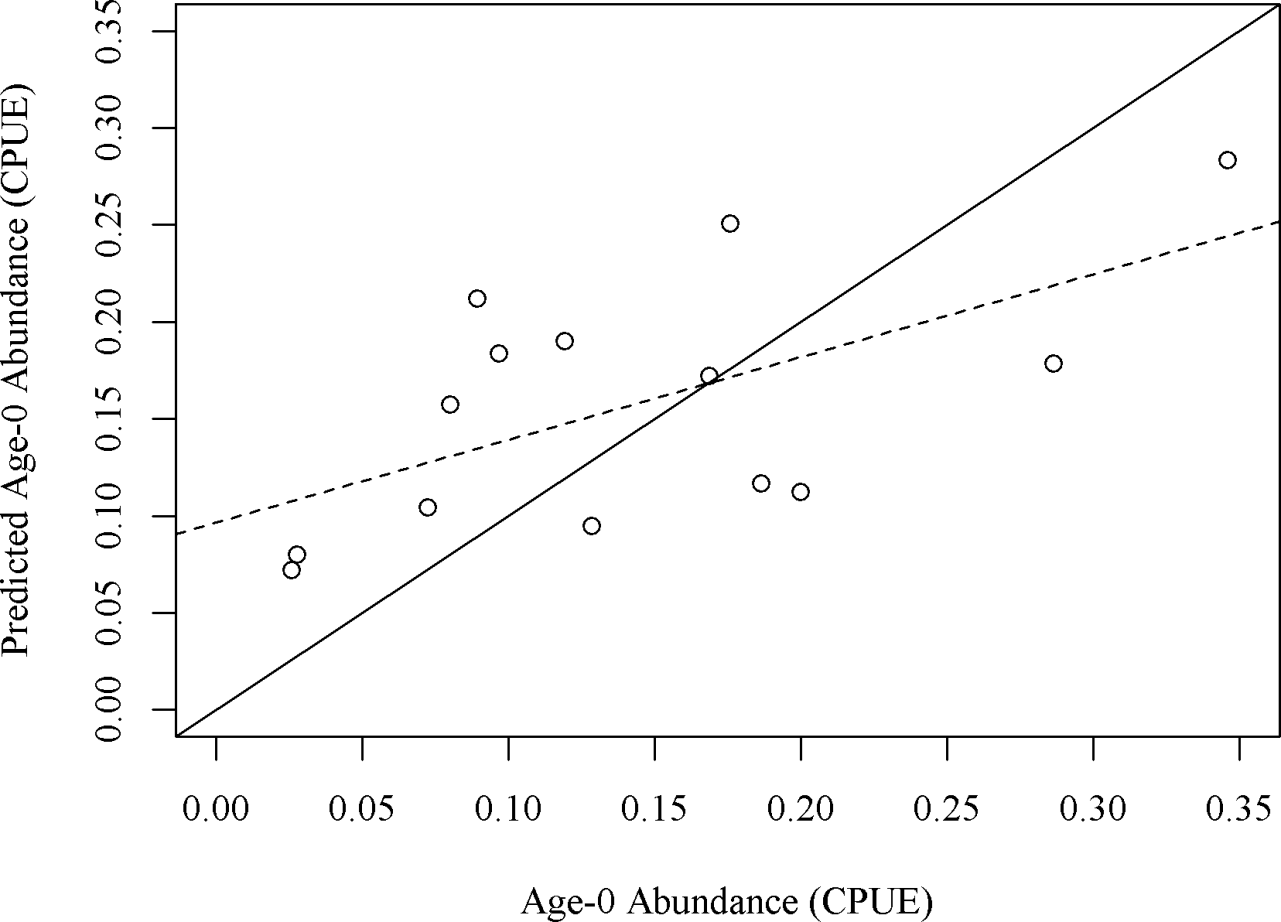
Relationship between empirical and predicted age-0 smallmouth bass abundance. Plot of age-0 smallmouth bass for each sample predicted by the Ricker recruit-spawner model plotted against the actual number of age-0 fish collected. The solid line shows a 1:1 fit and the dashed line shows the least squares linear regression of the modeled and observed data (p = 0.004, R^2^ = 0.48).

We solved for the parameters a, b, c, and d using non-linear regression. The final model took eleven iterations to converge on a solution and had a residual standard error of 0.0916. We next used the jackknife method to calculate standard error for each parameter ([40]; S1 Table). We then plotted predicted CPUE of age-0 fish and actual CPUE of age-0 fish (Fig 4) and calculated fit with a least squares linear regression.

To evaluate uncertainty within the model, we conducted a sensitivity analysis on each of the parameters predicted by the non-linear regression where all other values were held constant (May temperature, Adult CPUE, and June discharge were set at the mean values from collected data). We modified each parameter by ± 25% and recorded the values of predicted age-0 CPUE (S1 Fig). The parameter modifying adult CPUE (b) was the most influential in the model and so we next predicted age-0 CPUE based on a range of adult CPUE values. For each adult CPUE value, we varied the parameter b ± 25% to demonstrate model uncertainty (Fig 4). All statistical analyses were conducted in R [38].

### Model parameters

Age-group mortality within the simulation model was assessed through two different mechanisms. First was natural mortality factors that varied among age groups in the simulation model (Table 2). Natural mortality values were based on previous work in the Buffalo River, but these studies only estimated survival for all ages combined and do not separate harvest mortality [33]-[34]. We modified these estimates to reflect higher mortality of young age classes (Table 2). Age-0 fish mortality is set at a low level to reflect overwinter mortality as age-0 fish abundance is predicted for October. We assigned a standard deviation to every mortality value to create variation among simulation years and reflect the stochastic nature of stream systems. The second form of mortality that we assessed was harvest mortality. We applied harvest mortality to adult fish age-4+ as the Buffalo River currently has a 305 mm length limit and most age-4+ smallmouth bass in the river are above this length cutoff [34]; AGFC unpublished age data). We estimated harvest mortality based on exploitation studies conducted by the Missouri Department of Conservation in similar Ozark rivers [21].

**Table 2:** Smallmouth bass age-structured model parameters. Each year in the simulations a random, normally distributed value for each parameter is selected based on the mean and standard deviation listed below.

Variable	Mean	SD
Environmental Parameters		
Present May Temperature	18.33°C	0.3
Future May Temperature	21.97°C	0.59
Present June Discharge	12.87 m^3^/s	8.84
Future June Discharge	11.83 m^3^/s	10.51
Minimum Discharge	0.5 m^3^/s	
Flood chance low	10%	
Flood chance high	20%	
Moderate/strong drought chance (present drought frequency)	34%; 14%	
Moderate/strong drought chance (future high drought frequency)	39%; 23%	
Mortality Parameters		
Age-0 Flood Mortality	0.9	0.01
Age-0 Natural Mortality	0.1	0.1
Age-0 Mortality Moderate Drought	0.3	0.1
Age-0 Mortality High Drought	0.5	0.1
Age-1 Natural Mortality	0.4	0.1
Age-1 Mortality Moderate Drought	0.6	0.1
Age-1 Mortality High Drought	0.8	0.1
Age-2 Natural Mortality	0.3	0.1
Age-2 Mortality Moderate Drought	0.45	0.1
Age-2 Mortality High Drought	0.6	0.1
Adult Natural Mortality	0.3	0.1
Adult Mortality Moderate Drought	0.45	0.1
Adult Mortality High Drought	0.6	0.1
Adult Fishing Morality Low	0.2	0.05
Adult Fishing Morality High	0.35	0.05
Mortality Lower Limit (all ages)	0.05	
Mortality Upper Limit (all ages)	0.95	
Population Parameters		
Initial number of age-0 fish	1.2	0.2
Initial number of age-1+ fish	0.15	0.1

### Simulations

The simulation model runs on an annual time step from October to October and each simulation was conducted over 100 years and replicated 1,000 times. We began each simulation with a very high abundance for each age group of fish (Table 2). A break-in period followed before the model settled into a relatively stable abundance of each age group of fish around year ten. Preliminary runs of our simulation model indicated an under prediction of adult abundance in present climate simulations compared to empirical data. Therefore, we modified the number of age-0 smallmouth bass predicted by the Ricker recruit-spawner model by multiplying by four in all simulations. This resulted in very similar predicted abundances in the present climate simulation to the mean CPUE of adult smallmouth bass collected in empirical data.

We conducted ten different simulations where we examined different climate and harvest related scenarios. The first simulation was based on present climate conditions where June discharge and May temperature were taken from historical values for the Buffalo River area. Using the NOAA climate station and USGS river gage described above, we calculated a mean value and standard deviations based on May temperature from the years 1948–2013, and a median value and standard deviation based on June discharge from the years 1940–2013. The remaining nine simulations were set at future climate conditions for June discharge and May temperature. We determined future mean monthly May temperature and mean monthly June discharge values based on climate simulation results from an ensemble average of 30 downscaled climate models for an RCP 8.5 emissions scenario at mid-century (2050–2074; USGS National Climate Change Viewer; [12]). Mean minimum and maximum temperatures output by the climate models were averaged and used to determine a future mean monthly May temperature with standard deviation. Because the Buffalo River is runoff dominated [41], we determined future discharge by modifying historical median discharge proportionally to the change in future projected precipitation (the mean is about 8% lower in June and 19% more variable).

We modeled two different types of drought simulations based on current and future projected drought frequency in the region. Summer drought conditions are presently common in runoff dominated rivers like the Buffalo River in this region [29]-[30], [41] and climate change could lead to prolonged and more severe drought conditions in the region [26]. We modeled drought probabilistically where each simulated year had a chance of being a drought year (Table 2). We based present drought chances on long term discharge data from the Buffalo River during summer months (June-September) where we defined a moderate drought year as being lower than 50% of mean discharge during the period of record (1940–2013) and a strong drought year as being less than 25% of mean discharge. Natural mortality during moderate drought years was increased 50% above normal levels for all age groups and natural mortality during strong drought years was increased 75% above normal levels for all age groups. In future higher frequency drought simulations, we modeled expected future increases in drought in this region (moderate drought 5% more frequent and extreme drought 9% more frequent; [26]).

We modeled two different types of flood simulations based on current and future projected flood frequency in the region. We simulated flooding during the spawning and rearing period and the associated mortality of age-0 fish. Though floods likely affect older age groups as well, it is more difficult to quantify those effects. Similar to drought, we simulated flooding probabilistically where every year had a chance to be a flood year. We based present flood probability on discharge data from the Buffalo River (1940–2013) during June where we defined a flood event as an increase in mean June discharge of 100% above median discharge (about 10% of years from 1940–2013). If a flood year occurred, mortality of age-0 fish was set at 90%. We modeled some scenarios as having a higher flood chance to simulate an increase in extreme precipitation events due to climate change. Though increases in extreme precipitation events are expected in this region, there are no models projecting the associated effects on numbers of flood events with high confidence in this region [42]. We chose to increase flood probability in a given year to 20% as an exploratory examination of future flooding potential.

We modeled two different harvest morality simulations. Though recent estimates of harvest of smallmouth bass in the Buffalo River are not available, we used harvest mortality estimates from similar Missouri Ozark rivers in our simulations (approximately 20% annual harvest mortality; [21]). Use of the Buffalo River has increased in recent years (a 240% increase in visitors from 2000 to 2016; [43]) and is likely to increase in the future, potentially leading to higher harvest of smallmouth bass. Assuming no change in regulations and an increase in river use, we modeled a future harvest scenario as 75% higher than present harvest mortality (35% annual harvest mortality). Finally, we modeled a scenario where harvest of smallmouth bass is closed to facilitate a comparison of the importance of harvest and other mortality factors in affecting population levels.

## Results

### Empirical data and Ricker recruit-spawner model

A total of 325 age-0 smallmouth bass and 994 adult smallmouth bass were retained for use in analyses. Catch per unit effort ranged from 0.03 to 0.41 for age-0 fish and from 0.11 to 0.99 for adult fish. Age-0 CPUE was most strongly related to May temperature (R^2^ = 0.15) and June discharge (R^2^ = 0.29) and these variables were included in the Ricker recruit-spawner model.

**Fig 5:**
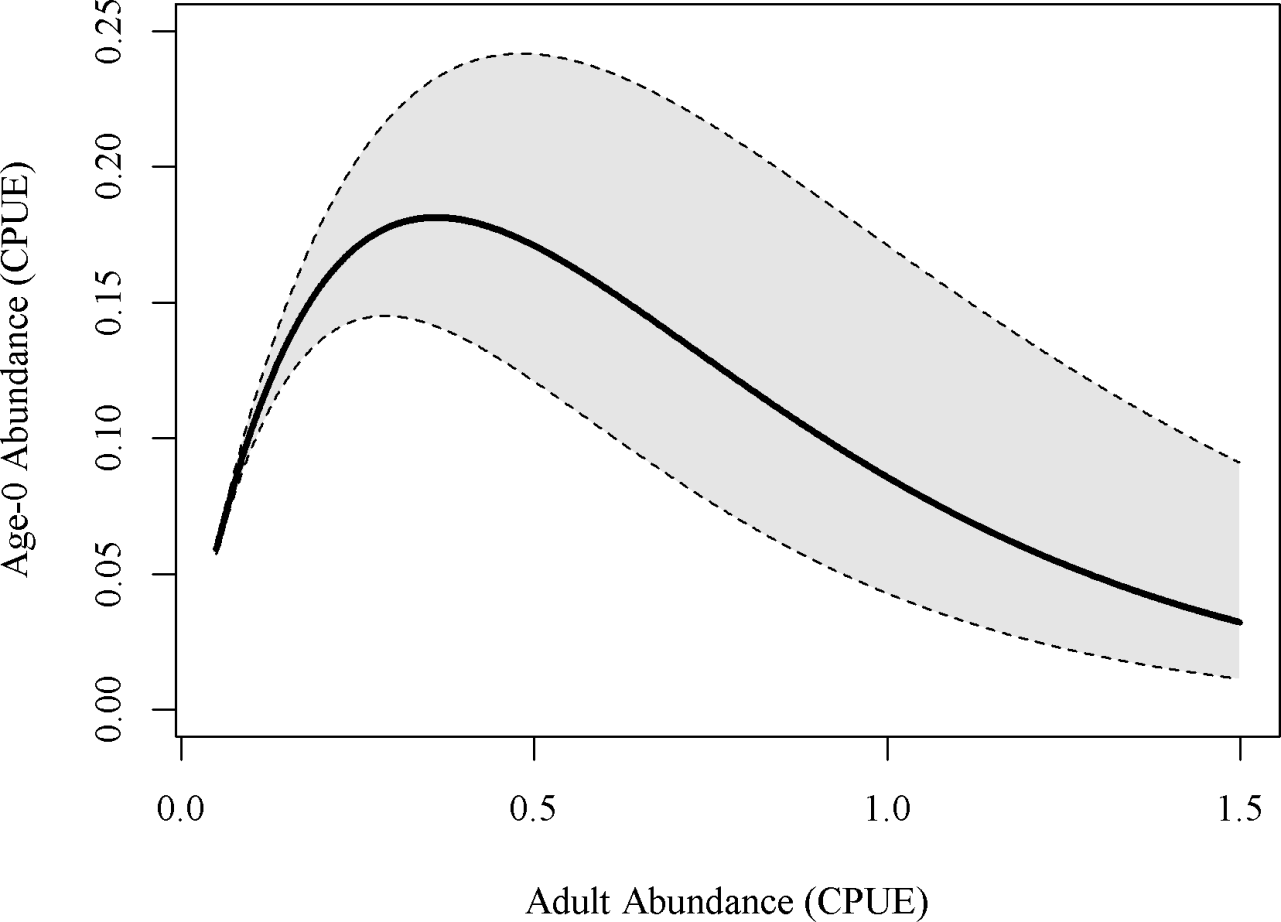
Ricker recruit-spawner model age-0 fish abundance prediction. Here the number of age-0 fish is predicted at varying levels of adult abundance. All parameters are held at a constant value except for adult abundance. Grey area shows the range of values for adult abundance as the parameter b is varied ±25%.

Sensitivity analysis was performed on the four parameters within the Ricker model equation (S1 Fig). These analyses indicated that parameter b, modifying adult abundance, was the most influential parameter in the model. A ± 25% change in b elicited a 39% decrease and a 29% increase in CPUE of age-0 fish predicted by the model when all other parameters were held at mean values. A ± 25% change in a resulted in a 13% decrease and a 15% increase in CPUE of age-0 fish. A ± 25% change in c resulted in a 10% decrease and a 12% increase in CPUE of age-0 fish. A ± 25% change in d resulted in a 16% decrease and a 19% increase in CPUE age-0 fish. Because of the indicated importance of adult abundance in structuring model results, we plotted predicted age-0 abundance by a range of adult abundance values to examine model behavior with the error ranges showing the range of results if b was varied ± 25% (Fig 5). The linear regression between predicted age-0 CPUE and observed age-0 CPUE indicated that our model adequately predicted the number of age-0 fish (p = 0.004, R^2^ = 0.48).

### Simulation results

**Fig 6:**
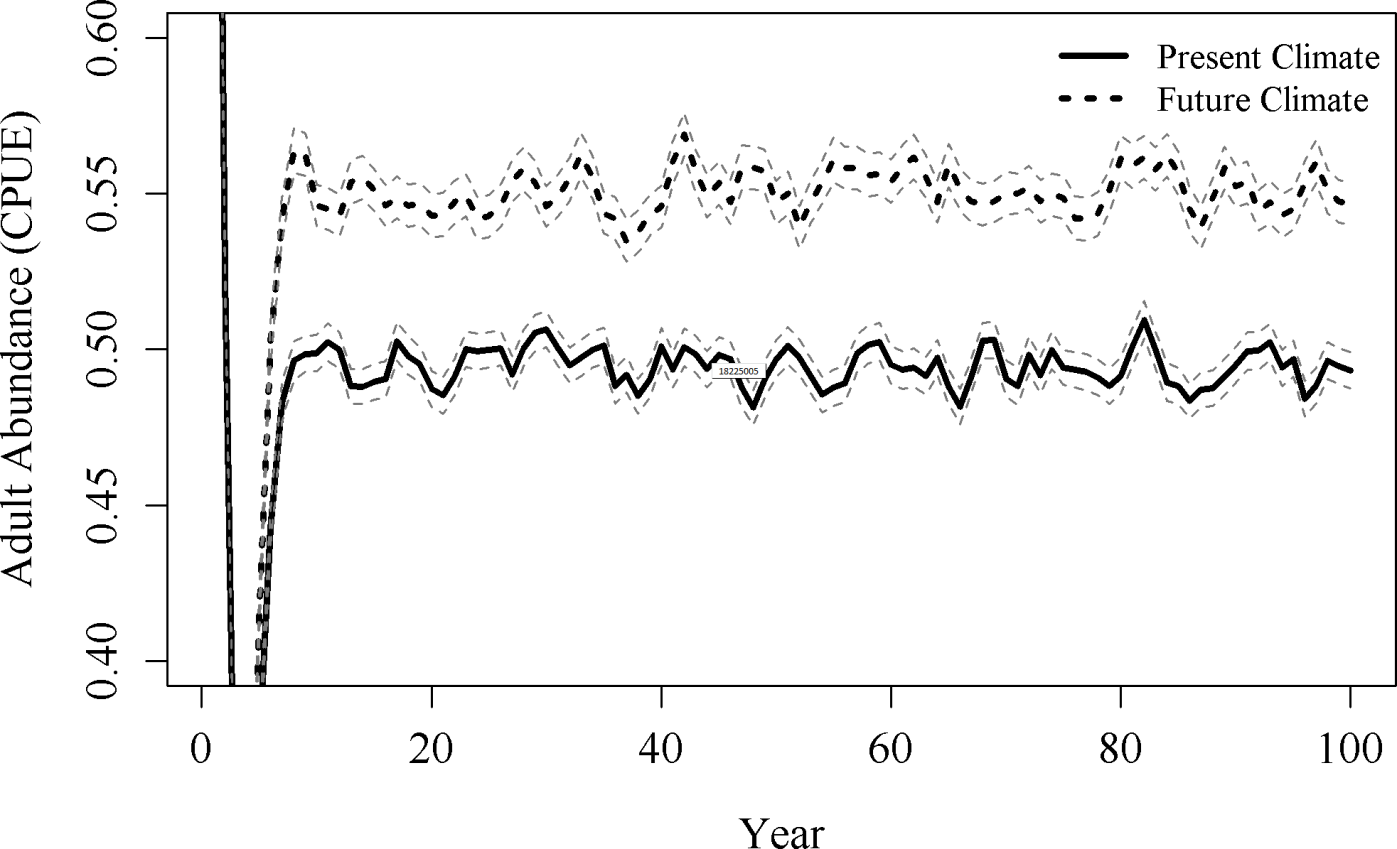
Simulation model results for present and future conditions. Mean adult abundances from the 1,000 replications of the present (solid black line) and future climate (dashed black line) simulations. Grey dashed lines represent standard error.

**Fig 7:**
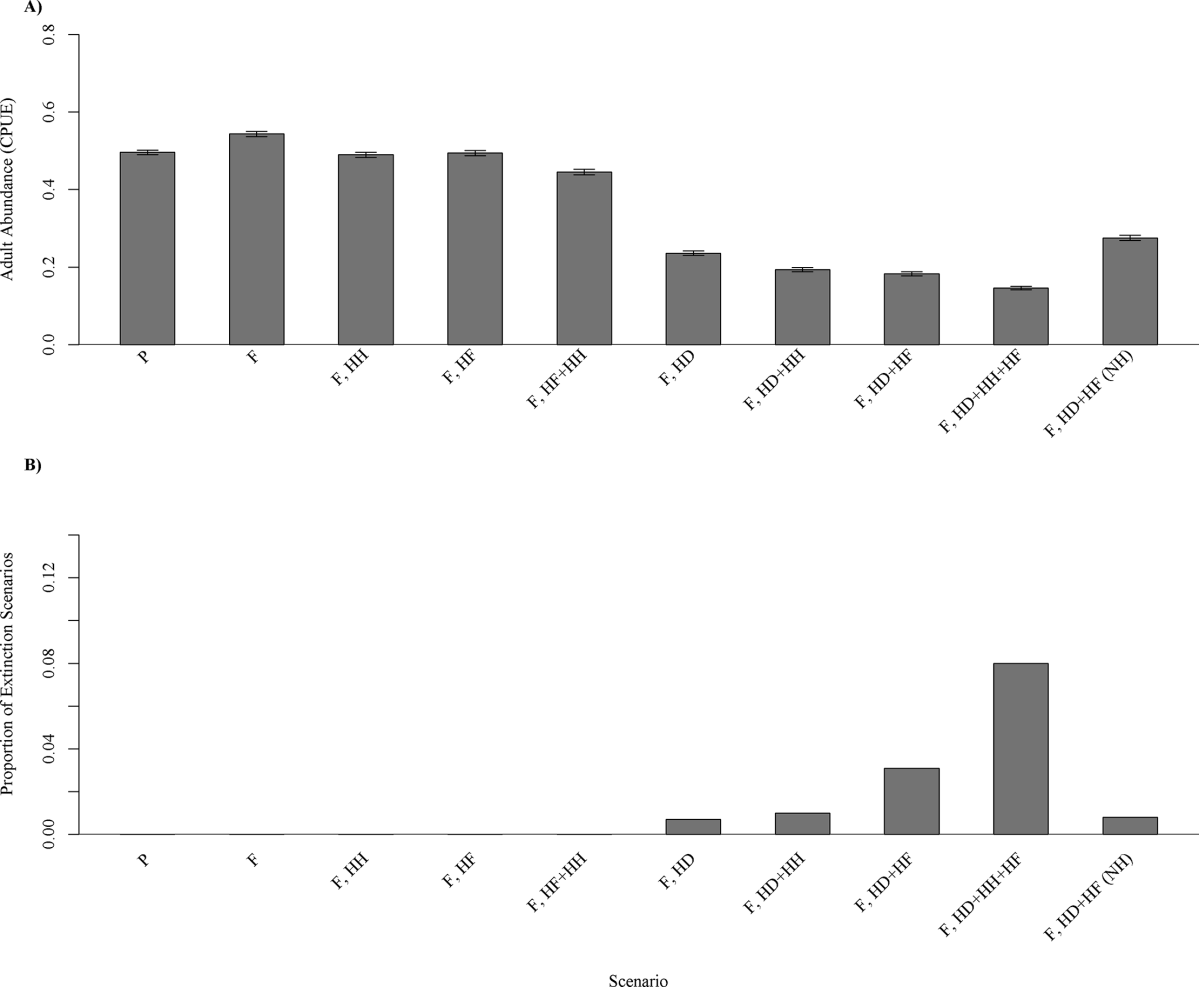
Results for all simulation model scenarios. Mean adult abundances based on 1,000 replications for the final year (year 100) for each model simulation (A) and proportion of simulations where smallmouth bass population extinction occurred (B). P represents present climate conditions (May temperature and June discharge), F represents future climate conditions (May temperature and June discharge), HH represents high harvest, HF represents high flood, HD represents high drought, and NH represents no harvest. Error bars indicate standard error based on the 1,000 replications of each simulation.

The future climate simulation predicted a 15% increase in abundance of adult smallmouth bass compared to present conditions (Fig 6). This scenario only took into account changes in May temperature and June discharge, not increased flooding, drought, or harvest conditions. The only other scenario that predicted an increase in abundance of adult smallmouth bass from present conditions was the future high flood scenario (6% increase in adult CPUE; Fig 7). Simulations with increased drought chances predicted a large decline in adult abundance from present conditions (52% decline in adult CPUE for the high drought scenario; Fig 7A) and the scenario with high drought, high flood, and high harvest had the greatest change from present conditions for any modeled scenario (71% reduction in adult CPUE; Fig 7A). In the final scenario, harvest was eliminated in the model, resulting in a 46% decline in CPUE compared to the present climate simulation, less of a decline than any other scenario with high drought included (Fig 7A). The only scenarios where extinction occurred were scenarios that included high probabilities of drought. Extinction probabilities ranged from 0 in present climate conditions to 0.08 in the scenario with high drought, flood, and harvest (Fig 7B).

## Discussion

Our simulation model predicted an increase in abundance of adult smallmouth bass under future May temperature and June discharge conditions, but this population increase could be offset by other expected climate related changes in the region such as increased drought and flooding. Temperature and discharge during the spawning and rearing period have been shown to affect year-class strength of lotic smallmouth bass in other streams [5], [13], [35]-[37]. Mean June discharge was found to be the best predictor of smallmouth bass recruitment in three Virginia Rivers and years with very high flows in June led to near year-class failures [5]. Similarly, several sportfish species were negatively related to spring season discharge in four Florida rivers [44]. Higher temperatures during spring and summer months can positively affect smallmouth bass recruitment through increased growth which can reduce predation risk and increase overwinter survival [45]. Climate change is likely to result in an increase in May temperature and a decline in June discharge, both of which should benefit smallmouth bass recruitment, leading to higher adult abundances.

Flooding during the spawning and rearing period can devastate year classes of lotic smallmouth bass [5] and other lotic black bass species (e.g., shoal bass, [7]). Heavy precipitation events have increased over the past century, leading to more frequent high flow events [46]. Climate change is likely to increase the frequency of severe storms that will affect short-term discharge variation [47] and lead to more high flow events. However, high flow during any period of spawning and rearing is likely to negatively affect smallmouth bass year class strength [13]. This corresponds to our empirical data where a single year had a very high June discharge level (23.28 m^3^/s) and almost no age-0 smallmouth bass were collected that year. Overwinter discharge can also be an important predictor of smallmouth bass recruitment [13]. We chose not to include overwinter discharge or temperature in the juvenile fish reproduction model because overwinter survival is less of a concern at southern latitude populations as spawning occurs relatively early in the year, allowing time for a long growing season before winter [48]-[49].

Our simulations indicate that increases in drought frequency could strongly affect abundance of smallmouth bass in the Buffalo River. Previous work has documented a decline in body condition of smallmouth bass during summer months in some streams in the Ozark region, including the upper Buffalo River [50], and it is likely that increasing stream temperature due to climate change will decrease growth potential of smallmouth bass during summer months [28]. Drought conditions can stress fish and result in population declines [51]. Drought in the Ozark-Ouachita Interior Highlands leads to pool isolation in runoff streams and pool isolation can increase competition and predation risk [52]-[53]. Strong drought can cause severe abiotic conditions within pools [54] and can lead directly to mortality of smallmouth bass through complete drying of pools [30]. In our simulations, drought was the only stressor that affected every age class, leading to the strong effects we found.

Angler harvest is an important mortality component in exploited fish populations. A recent angler creel survey on the Buffalo River found almost no harvest of smallmouth bass, despite high levels of angler effort [55]. In contrast, work conducted in six Ozark streams in Missouri found that exploitation ranged from 7–26% of the population with three rivers having exploitation rates over 20% [21]. We chose to model current exploitation near the high level found in Missouri streams as the Buffalo Creel survey authors urge caution in utilizing the estimates from the creel survey [55] and the Buffalo River likely experiences as high or higher usage than the Missouri streams [43]. Missouri Ozark streams are closed to harvest for approximately three months in the spring, but there is no harvest closure in the Buffalo River, indicating that our present simulation harvest estimate is likely conservative. Our high exploitation scenario reflects increasing usage of the river in future years.

There are several limitations inherent in our modeling approach. We do not attempt to model some aspects of smallmouth bass population dynamics such as growth, intraspecific competition, migration, and dispersal. Our modeling also only takes into account a few of the potential effects of climate change. For example, we do not simulate habitat or land use changes that could occur over the coming century. However, a riparian buffer around the Buffalo River is protected by the National Park Service which may limit effects of land use change on the river. We also assume that relationships between May temperature and June discharge and smallmouth bass recruitment will be the same in the future as during the period that data was collected. The Buffalo River is a runoff stream and other stream types, such as intermittent or groundwater dominated, could have different responses to climate change than runoff streams (e.g., temperature, [28]). Another simplifying assumption occurs in the way we model harvest mortality. We do not take into account catch and release mortality that can be an important component of overall mortality [56]. In our closed harvest scenario in particular, catch and release mortality or sub-lethal effects would likely still occur, especially at high temperatures during summer months [57], which could reduce the effectiveness of eliminating harvest through changing regulations. An additional assumption of our model is that variables such as age-specific mortality vary independently each year.

Our model has a number of management implications. Our results indicate that increases in drought prevalence could have the strongest effects on smallmouth bass abundance. Management actions designed to mitigate the effects of drought could reduce these effects. For example, management in this region could be directed towards reducing water withdrawals [58], preserving spring inputs into streams, and increasing riparian canopy to reduce water temperatures and evapotranspiration [27]. Though we found that increases in flooding frequency had a much smaller effect on adult smallmouth bass abundance than drought, reducing flooding risk by decreasing channelization and protecting the riparian buffer could reduce the flashiness of streams [59]. The Buffalo River region is remote, reducing the risk of major land use changes over the coming century, but increases in development and agriculture usage in the watershed could further increase flooding chances and alter discharge during the spawning and rearing period, affecting fish populations [13].

More restrictive harvest regulations could reduce the negative effects of climate change on smallmouth bass populations in the Buffalo River. Currently, the river has a twelve inch minimum length limit and a four fish bag limit. In future climate scenarios with high drought and high flooding probability, smallmouth bass populations were 52% lower in scenarios with high harvest and 30% lower in scenarios with present harvest levels as compared to a simulation without harvest included. In addition, removing harvest almost eliminated the chance of smallmouth bass population extinction occurring. Managers could consider restricting or eliminating bag limits for smallmouth bass if noticeable declines in adult smallmouth bass abundance occur due to climate change.

## Conclusions

We found that changes in May temperature and June discharge could benefit smallmouth bass recruitment, but increased flooding and increased drought conditions are likely to reduce adult smallmouth bass abundance below present levels in the Buffalo River. Reducing or eliminating harvest could prove a viable strategy to reduce the negative effects of climate change and lessen the risk of population extinction. Efforts to reduce flooding or drought effects within the Buffalo River could also reduce the negative effects on smallmouth bass populations. Future work is needed to further understand the effects of climate change on smallmouth bass populations at the southern range extent, especially in streams from differing flow regimes.

## Supporting information

S1 Table Standard errors for parameters from the Ricker recruit-spawner model. Standard errors are calculated using the jackknife method.
(DOCX)

S1 Fig Sensitivity analysis results. Each parameter was varied ±25% of the value solved for in the non-linear regression. As each parameter was tested, all others were held at the solved value and other model data (adult abundance, May temperature, June Discharge) were held at mean values based on the original data.
(TIF)
