# Supplementary material for: Forecasting effects of angler harvest and climate change on smallmouth bass abundance at the southern edge of their range
Source: PLoS One. 2018 Aug 20;13(8):e0202737. doi: 10.1371/journal.pone.0202737 (PMC6101403; doi:10.1371/journal.pone.0202737)
Supplement: S1 Table — Standard errors are calculated using the jackknife method. (DOCX) [file pone.0202737.s001.docx]

| Parameter | Value | Standard Error |  |  |
| --- | --- | --- | --- | --- |
| A | 0.570 | 0.488 |  |  |
| B | 2.767 | 0.205 |  |  |
| C | 0.023 | 0.023 |  |  |
| D | -0.064 | 0.006 |  |  |
