# Supplementary figures and images for: Forecasting effects of angler harvest and climate change on smallmouth bass abundance at the southern edge of their range
Source: PLoS One. 2018 Aug 20;13(8):e0202737. doi: 10.1371/journal.pone.0202737 (PMC6101403; doi:10.1371/journal.pone.0202737)

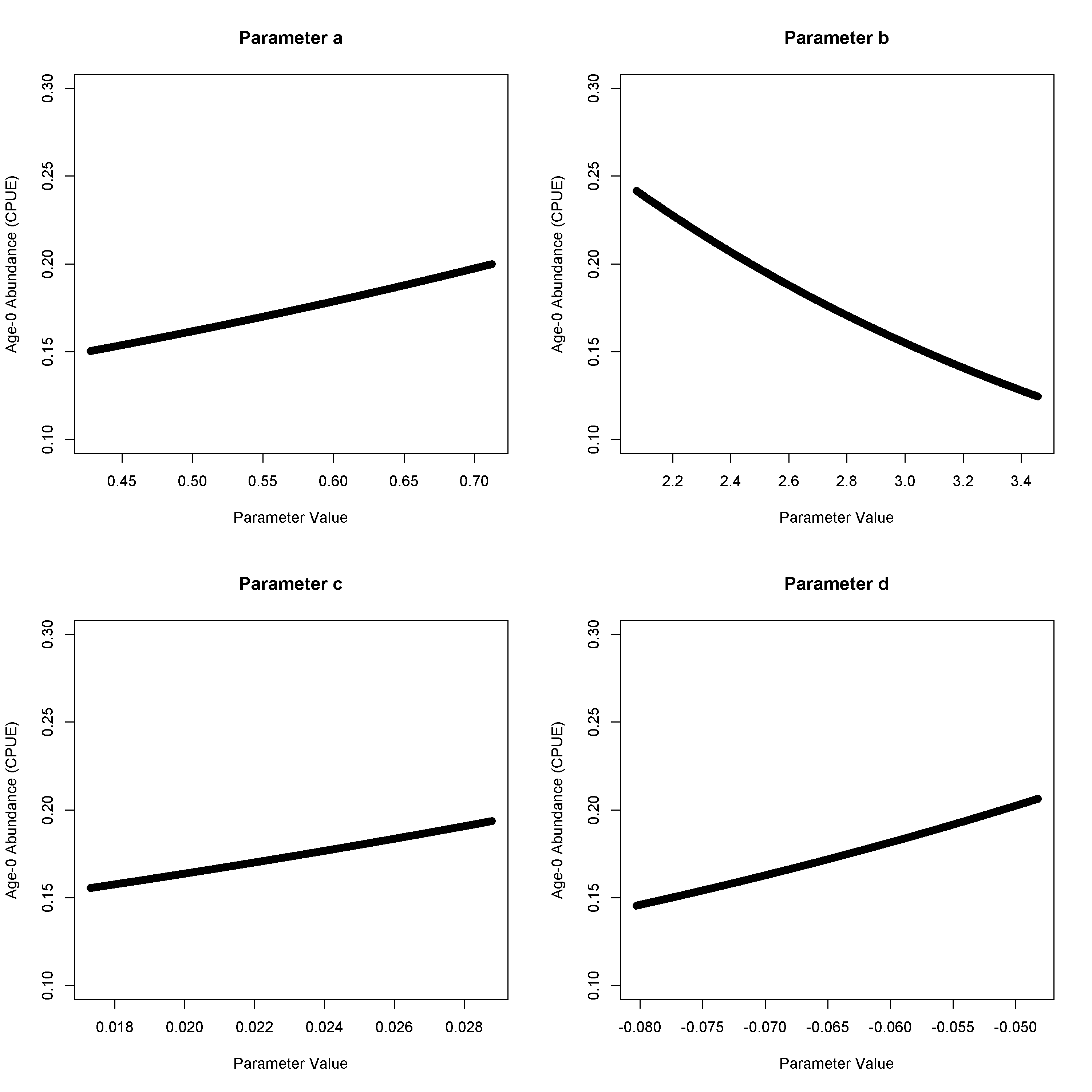

Supplement: S1 Fig — Each parameter was varied ±25% of the value solved for in the non-linear regression. As each parameter was tested, all others were held at the solved value and other model data (adult abundance, May temperature, June Discharge) were held at mean values based on the original data. (TIF) [file pone.0202737.s002.tif]
